# *KAT6A::EP300* fusion in congenital myeloid sarcoma: Yet another novel molecular marker indicating spontaneous remission?: A case report

**DOI:** 10.1097/MD.0000000000034258

**Published:** 2023-07-28

**Authors:** Smitha Hosahalli Vasanna, Sonal D. Shah, Bethany R. Rohr, Breanne Roche, Howard Meyerson, Irina Pateva

**Affiliations:** a Department of Pediatrics, Division of Pediatric Hematology-Oncology, University Hospitals-Rainbow Babies and Children’s Hospital, Cleveland Medical Center, Cleveland, OH; b Department of Dermatology, University Hospitals Cleveland Medical Center, Cleveland, OH; c Department of Pathology, University Hospitals Cleveland Medical Center, Cleveland, OH.

**Keywords:** infant AML, *KAT6A::EP300* fusion, leukemia cutis

## Abstract

Acute myeloid leukemia (AML)/myeloid sarcoma (MS) is risk-stratified based on cytogenetics. Although most congenital AML/MS have a dismal prognosis, certain genetic variants such as t (8, 16) [*KAT6A::*cAMP response element-binding protein (CREB) - binding protein fusion] and more recently t (8, 22) [*KAT6A::EP300* fusion] have shown spontaneous remissions. *KAT6A* located on chromosome 8p11 encodes KAT6A protein, a histone/lysine acetyltransferase enzyme. Numerous partner genes associated with *KAT6A* include cAMP response element-binding protein (CREB) - binding protein (16p13), *EP300* (22q13), *LEUTX* (9q13), *NCOA2, NCOA3*, and *ASXL2*.

**Patient concerns:**

In this article, we describe an otherwise healthy infant who presented with skin nodules on the face and scalp without any systemic or CNS involvement. A biopsy of the cutaneous lesion was consistent with congenital MS.

**Diagnoses:**

Through molecular testing, we found that our patient had the *KAT6A::EP300* mutation. This is one of the rare recurrent cytogenetic abnormalities that are linked to congenital AML.

**Intervention:**

Our patient underwent spontaneous remission with watchful waiting.

**Outcome:**

Our patient has remained in spontaneous remission for 24 months.

**Lessons:**

Even though the *KAT6A::EP300* mutation in adults is a poor prognostic marker, a similar mutation in congenital AML has a higher likelihood of spontaneous remission. Hence, conservative management might be an initial management strategy for clinically stable patients.

## 1. Introduction

Congenital myeloid sarcoma (MS) of the skin has a variable clinical course, ranging from spontaneous remission to rapidly progressive disease, with systemic involvement based on the underlying molecular defect. Molecular subtypes with higher chances of spontaneous remission have been described with t (8; 16) [*KAT6A::*cAMP response element-binding protein (CREB) - binding protein (CREBBP) fusion].^[1,2]^ Only 1 known case of an infant with t (8;22) [*KAT6A::EP300* fusion] undergoing spontaneous remission has been reported in Japan.^[3]^ We report a case of congenital skin MS with *KAT6A::EP300* fusion, with sustained spontaneous remission for 24 months.

## 2. Case report

**Figure 1.**
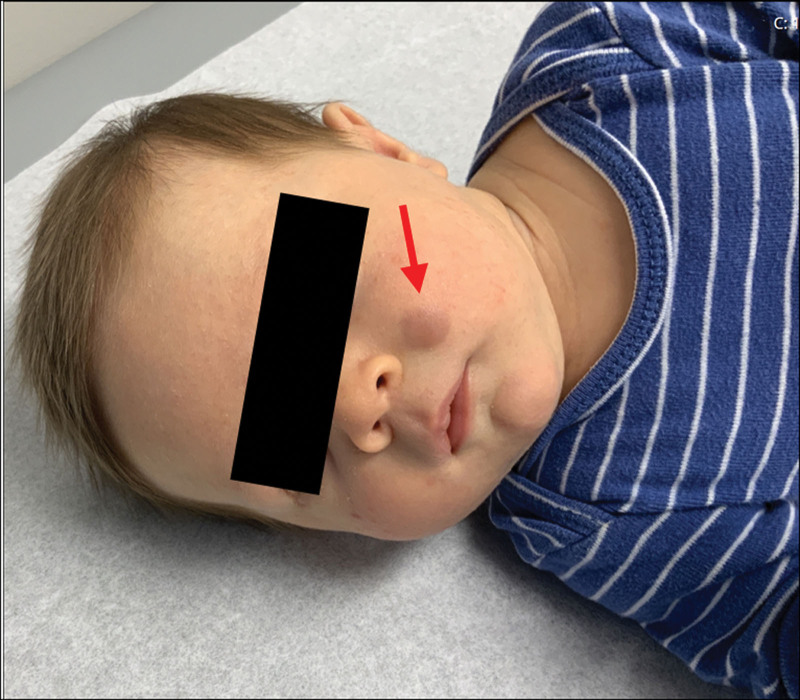
Left medial cheek with a firm blue to violaceous subcutaneous nodule with no overlying surface change.

**Figure 2.**
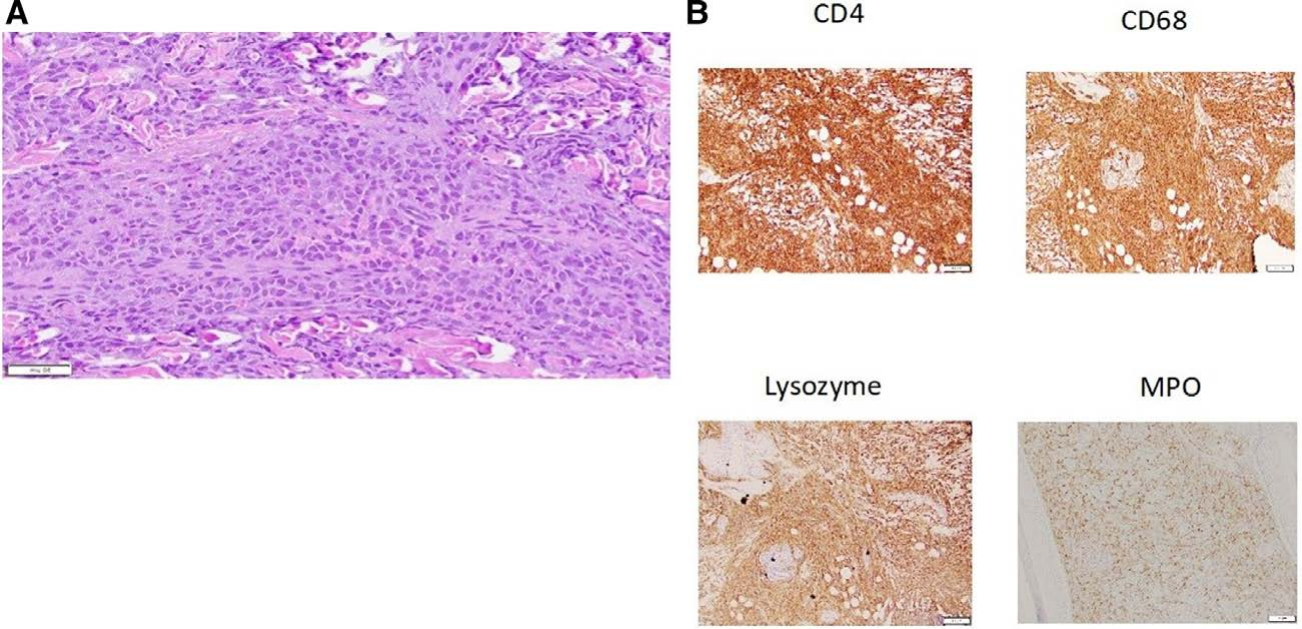
(A) Histopathology reveals and infiltrate of enlarged atypical and mitotically active neoplastic cells (H&E, 20X magnification). (B) Immunohistochemistry reveals CD4, CD68, and lysozyme positivity with partial positivity for myeloperoxidase (10x magnification) suggestive of myeloid sarcoma with monocytic features.

A 1-month-old male infant presented to the dermatology clinic with a slow-growing, bluish-purple firm nodule on his left cheek (Fig. 1) noticed at 2 weeks of age. He was otherwise in good health and met age-appropriate growth and developmental milestones. His antenatal and postnatal histories were unremarkable. At 6 weeks of age, a similar new nodule was noted on the scalp. The remainder of his physical examination results were unremarkable. Ultrasound of the cheek lesion demonstrated a subcutaneous soft tissue mass with mildly increased flow, measuring 1.5 × 1.2 × 0.6 cm. A broad differential diagnosis was considered, including deep infantile hemangioma, cutaneous neuroblastoma, Langerhans cell histiocytosis, extramedullary hematopoiesis, and rhabdomyosarcoma. The scalp nodule was biopsied, and histopathology revealed dense infiltration of the dermis and subcutaneous fat by blastic mitotically active neoplastic cells (Fig. 2A). Immunohistochemistry was uniformly positive for CD4, CD68, and lysozyme and variably positive for myeloperoxidase, but negative for CD3, CD20, CD34, CD56, CD117, CD123, CD1a, AE1/AE3, CK20, desmin, S100, and Leder, suggesting myeloid sarcoma with monocytic features (Fig. 2B). The infant was referred to the Pediatric Hematology Oncology Department.

Initial laboratory work revealed normal peripheral blood counts (Hemoglobin: 10.7 g/dL, white blood cell count, 12,000 × 10^9^/L; platelets, 430,000 × 10^9^/L with a normal differential), normal serum chemistries and tumor lysis labs. Workup for systemic involvement, including bone marrow aspiration/biopsy (both by morphology and flow cytometry) and positron emission tomography–computed tomography, was negative. Bone marrow cytogenetics were negative for any acute myeloid leukemia (AML) related genetic mutations. Lumbar puncture was not performed because of the absence of systemic involvement. A skin biopsy of the cheek lesion also confirmed the presence of abnormal monocytic cells, from which the tissue was sent for cytogenetic analysis. Fluorescence in situ hybridization was negative for *KMT2A*, inv (16), t (8; 21), and t (8; 16) translocations. FoundationOne CDx testing revealed a t (8;22) (p11; q13) translocation with resultant *KAT6A::EP300* fusion, which has been associated with spontaneous remission. A *GATA2* P161A mutation was also present but was reported as a variant of unknown significance. Given that the identified mutation has been reported to have spontaneous remission in infants, along with the young age of our patient and risks of systemic therapy, a decision for observation was agreed upon by the patient’s family. The patient has been in remission for 24 months. He was monitored with monthly physical examinations and laboratory evaluations in the first year after diagnosis and every 3 to 4 months thereafter. There was only a small visible scar at the biopsy site on his left cheek, and the scalp lesion completely resolved. As there was no bone marrow involvement at diagnosis, no repeat testing was performed.

## 3. Discussion

Congenital MS is characterized by the accumulation of myeloid blasts at extramedullary sites, the most frequent sites being the skin (called leukemia cutis) and soft tissues, where it can present as blue to violaceous, firm subcutaneous papules and nodules.^[4]^ It can be concurrently associated with bone marrow disease or be a precursor for systemic AML, with a usual time to progression of 4 months; hence, in most instances, systemic chemotherapy is required.^[5]^ Since nearly more than half of congenital/infantile leukemia cases harbor high-risk cytogenetics such as *KMT2A* rearrangements, prognosis is considered dismal.^[6]^ Although the reported overall survival for congenital AML is as low as 25%, certain genetic variants, such as t (8;16) and more recently t (8;22), have been reported to have undergone spontaneous remissions.^[1–3,7,8]^

*KAT6A* (also referred to as *MYST3*) is located on chromosome 8p11 and encodes a histone lysine acetyltransferase protein called monocytic leukemia zinc finger protein.^[9]^ *KAT6A i*s a transcriptional coactivator that regulates the expression of hematopoietic genes by interacting with *RUNX1* and *SPI1/PU.1*.^[10,11]^ In addition, *KAT6A* and *KMT2A* regulate *HOX* genes in human cord blood CD34 + cells, which are epigenetic regulators of hematopoiesis.^[12]^

The most common fusion partner of *KAT6A* is *CREBBP, CBP*, which is located on chromosome 16p13.^[13]^ *KAT6A::CREBBP* fusion or t (8;16) in pediatric AML is a rare but recurrent and distinct genetic alteration characterized by an association with congenital AML, mainly the M4 and M5 FAB subtypes. It is associated with a higher incidence of leukemia cutis, disseminated intravascular coagulation, and hemophagocytosis, however can attain spontaneous remission in a subset of infants.^[14]^

*EP300* is another partner gene that undergoes fusion with *KAT6A* with the resultant fusion *KAT6A-EP300* or t (8; 22) (p11; q13). Both *EP300* and *CREBBP* are members of a family of transcriptional adaptor proteins that share a close structural and functional relationship to regulate transcription and facilitate cellular differentiation.^[15]^ In addition, P300 interacts with MDM2 to regulate the turnover of the tumor suppressor protein p53.^[16]^ The fusion protein likely exerts an oncogenic effect by interfering with the normal fusion of transcription factors, including nuclear receptors and the RUNX1 protein.

Other partner genes with *KAT6A*, such as *NCOA2* have been reported in invasive ductal breast carcinoma, prostate, bladder, and colon cancers and AML in mouse models, *NCOA3* with M5 AML, and *ASXL2* with therapy-related AML in a 6-year-old with M2 AML treated with etoposide.^[17–19]^ Coenen et al^[14]^ studied a large cohort of patients with *KAT6A::CREBBP* fusion (n = 63) and reported spontaneous remission in 7 patients, of whom 3 remained in continuous remission; the 5 year overall survival rate was 59%. Sramkova et al^[20]^ reported a neonate with *KAT6::LEUTX* fusion who rapidly deteriorated and succumbed to the illness despite initiation of systemic chemotherapy. This fusion has been reported to result in remission after intensive chemotherapy.

Ikawa et al^[3]^ from Japan reported a newborn girl with leukemia cutis and bone marrow disease (44% blasts) with a monocytic variant, who had the same translocation as in our patient. With spontaneous regression of the skin lesions, watchful waiting was implemented, and she achieved complete and deeper molecular remission at 3 months of age. This case was published in 2018 when the patient was 2 years old, with minimal residual disease remission without any chemotherapy. In contrast, adult AML patients with the same translocation have been reported to have a poor prognosis.^[21]^ The above-mentioned alterations involving *KAT6A* and its partner genes occur more frequently in infant AML and therapy-related AML. Since it is difficult to predict the disease course, a decision for watchful waiting or treatment can be clinically challenging and needs to be made on a case-by-case basis.

Our patient had isolated cutaneous MS in the absence of systemic disease and remained in remission 24 months after diagnosis without therapy.

## 4. Conclusion

This is the second known reported case of *KAT6A::EP300* fusion undergoing spontaneous remission in congenital AML or myeloid sarcoma. Both cases highlight that close observation and avoidance of systemic therapy in infants with AML and *KAT6A::EP300* mutation may be a prudent initial management approach.

## Acknowledgements

Mandy Neudecker, librarian, Rainbow Babies and Children’s Hospital/ University Hospital, Cleveland Medical Center.

## Author contributions

**Resources:** Breanne Roche.

**Supervision:** Howard Meyerson, Irina Pateva.

**Writing – original draft:** Smitha Hosahalli Vasanna.

**Writing – review & editing:** Smitha Hosahalli Vasanna, Sonal D. Shah, Bethany R. Rohr, Breanne Roche, Howard Meyerson, Irina Pateva.
